# NutriCubes: Nutritional and Sensory Quality of Fermented Cereal-Legume Snacks

**DOI:** 10.3390/foods15193487

**Published:** 2026-09-29

**Authors:** Eugenia Covaliov, Tatiana Capcanari, Oxana Radu, Cătălina Negoița

**Affiliations:** Department of Food and Nutrition, Technical University of Moldova, 9/9 Studentilor St., MD-2045 Chisinau, Moldova; tatiana.capcanari@toap.utm.md (T.C.); oxana.radu@sa.utm.md (O.R.); catalina.cerchez@toap.utm.md (C.N.)

**Keywords:** snack, lactic acid fermentation, oat-legume blend, antinutritional factors, in vitro protein digestibility, polyphenols, CATA, protein quality

## Abstract

NutriCubes is a novel baked, cube-shaped snack formulated from oat-legume flour blends (70:30) incorporating lactic-fermented lentil (O-L), chickpea (O-C), or pea (O-P) flours, developed to bridge the gap between nutritional quality and convenience in the plant-based snack market. Fermentation with a mixed lactic acid bacteria (LAB) starter (*Lactiplantibacillus plantarum*, *Lacticaseibacillus casei*, and *Lactobacillus delbrueckii* subsp. *bulgaricus*; 10^8^ CFU/g; 30 °C; 24 h) reduced phytate by 53.3%, 45.3%, and 26.5% and tannins by 38.5%, 45.8%, and 37.5% in lentil, chickpea, and pea flours, respectively. All three NutriCubes formulations delivered 19.12–19.66 g protein/100 g fresh weight (FW) and 10.72–12.60 g dietary fibre/100 g FW. Cereal–legume complementarity was reflected in a favourable amino acid balance, with estimated Protein Efficiency Ratios ranging from 1.510 (O-C) to 1.989 (O-L). O-L showed the highest total polyphenol content (6.28 ± 0.13 mg GAE/g dry matter (DM)) and antioxidant activity (72.9 ± 1.22% DPPH inhibition). Hedonic panel evaluation (*n* = 107) confirmed strong acceptance across all formulations (7.48–8.12/9), with O-L scoring highest for taste (8.36 ± 0.70), colour (8.76 ± 0.44), and overall liking. The oat–lentil formulation emerged as the most nutritionally dense and sensorially preferred variant, establishing NutriCubes as a feasible snack with functional potential as an alternative.

## 1. Introduction

Snacking has become a defining feature of modern dietary behaviour. As lifestyles accelerate and meal patterns shift, between-meal consumption now accounts for a substantial fraction of daily energy intake in both developed and developing countries [1,2]. Yet the products that dominate the snack market—crackers, chips, puffed cereals—tend to be nutritionally thin: energy-dense, rich in refined starches and saturated fat, and poor in protein, fibre, and micronutrients [3]. Emerging evidence from observational studies and narrative reviews suggests an association between frequent consumption of ultra-processed snack foods and an increased risk of type 2 diabetes and cardiovascular disease [4,5]. Growing consumer awareness of this link has generated genuine market demand for alternatives that do not require people to compromise between convenience and nutritional quality [6].

Among the available raw material options, the combination of cereals and legumes stands out as particularly promising—not least because both ingredient groups are globally accessible, affordable, and already embedded in food cultures worldwide. Their pairing is scientifically compelling because of well-established nutritional complementarity: legumes such as lentils, chickpeas, and peas are rich in lysine and dietary fibre but are limited in sulphur-containing amino acids, whereas cereals like oats, corn, and rye supply methionine and cysteine but are limited in lysine [7,8]. Blending the two improves the indispensable amino acid profile, yielding amino acid score (AAS) values that more closely approach the FAO/WHO/UNU adult reference pattern [9,10,11]. Oats add an additional dimension through their β-glucan content, with documented effects on glycaemic response and blood cholesterol [12]; lentils, beyond their protein density, deliver a fibre and lysine profile that few plant ingredients match [13]. The formulation challenge, however, is real. Legumes carry a suite of antinutritional factors—phytic acid, tannins, trypsin inhibitors—that bind minerals, reduce protein digestibility, and can cause gastrointestinal discomfort [14,15]. Lactic acid fermentation, applied as a pre-treatment with strains such as *Lactiplantibacillus plantarum* (*L. plantarum*) and *Lacticaseibacillus casei* (*L. casei*), has been proposed as an effective approach to address this challenge. The metabolic activity of lactic acid bacteria lowers substrate pH, which is thought to activate endogenous phytases and facilitate the disruption of protein-antinutrient complexes; tannin-degrading enzymes produced by certain lactic acid bacteria (LAB) strains may additionally contribute to tannin reduction. Furthermore, the acidic environment generated during fermentation promotes the solubilisation of phytate-mineral-protein complexes, releasing bound minerals and improving protein accessibility to digestive enzymes. These mechanisms, however, are highly strain-, substrate-, and condition-dependent, and reported reductions in phytate and tannin content vary considerably across studies, ranging from 30 to 70% depending on fermentation parameters [15,16,17]. A reduction in the beany off-notes that typically limit consumer acceptance of legume products is an additional, practically important consequence of lactic fermentation, achieved primarily through the degradation of aldehydes such as hexanal—key contributors to the characteristic beany aroma impression—although the extent of this effect is highly dependent on the legume substrate, LAB strain, and fermentation conditions [18,19].

Research on legume-enriched snacks has grown substantially over the past decade. Patil et al. [20] showed that incorporating lentil and chickpea flours into wheat-based extrudates improved in vitro protein digestibility by 37–62%, even at substitution levels as low as 5–15%. López-Martínez et al. [21] went further, producing legume-only chips from broad bean and soybean blends that achieved 34.17 g/100 g protein and dietary fibre content more than twice that of conventional snacks. More recently, Mnayer and Joubrane [6] demonstrated that potato-based snacks enriched with soy and lentil protein flours at a 50:50 ratio could meet the ‘source of protein’ threshold under Codex labelling standards, and Ziena and Ziena [22] confirmed that complementing corn flour with faba bean and chickpea significantly improved essential amino acid scores alongside in vitro protein digestibility. What remains largely unexplored is the baked, geometrically defined snack format—and, more specifically, how blend diversity and fermentation pre-treatment interact across nutritional, bioactive, and sensory dimensions within a single product concept. Unlike previously reported formats, which typically fix the legume identity and vary only the substitution level within extruded or fried matrices, NutriCubes are produced by hot-water-induced starch gelatinisation and oven drying—a baked process that avoids high-temperature oil-assisted cooking—and apply lactic acid fermentation as a systematic, mandatory pre-treatment across three distinct legume species simultaneously, enabling direct comparison of how blend diversity modulates nutritional, bioactive, and sensory outcomes within a single product concept.

To the best of our knowledge, this is the first study to apply lactic acid fermentation independently to three distinct legume flours—lentil, chickpea, and pea—under identical, standardised conditions and to track the cumulative nutritional, bioactive, and sensory consequences through to a finished baked oat-legume snack, with CATA-based consumer evaluation, designated NutriCubes.

## 2. Materials and Methods

### 2.1. Materials

Oat flour (*Avena sativa* L.), lentil flour (*Lens culinaris* Medik.), chickpea flour (*Cicer arietinum* L.), and pea flour (*Pisum sativum* L.) were purchased from local commercial suppliers in Chișinău, Republic of Moldova, in their commercially milled forms. Legume flours were additionally ground in a laboratory knife mill (IKA A11 basic, IKA-Werke, Staufen, Germany) and sieved through a 250 µm mesh. Auxiliary ingredients—dried onion, dried garlic, mustard powder, refined sunflower oil, and food-grade salt—were sourced from the same retail network and maintained at fixed levels across all formulations.

The lactic acid fermentation was carried out using three LAB strains from established culture collections: *Lacticaseibacillus casei* ATCC 393 and *Lactiplantibacillus plantarum* subsp. *plantarum* ATCC 14917 (Microbiologics™, St. Cloud, MN, USA), and *Lactobacillus delbrueckii* subsp. *bulgaricus Lb14* (Thermo Fisher Scientific, Waltham, MA, USA).

### 2.2. Lactic Fermentation of Legume Flours

Prior to the NutriCubes formulation, each legume flour was subjected to a rapid lactic acid fermentation protocol. The flour was first hydrated with distilled water at a flour-to-water ratio of 1:5 (*w*/*v*) and conditioned at 35 °C for 45 min—a tempering step that activates endogenous phytases and creates favourable conditions for subsequent microbial fermentation, as the aqueous environment at a moderate temperature augments the natural phytolytic capacity of legume flours and enhances the ability of lactic acid bacteria to further degrade phytate [15,23].

The conditioned slurry was then inoculated with a mixed starter culture comprising *Lactiplantibacillus plantarum*, *Lacticaseibacillus casei*, and *Lactobacillus delbrueckii* subsp. *Bulgaricus*, combined in equal proportions (1:1:1, *v*/*v*), at a combined inoculation level of 10^8^ CFU/g [19,24]. Fermentation was carried out at 30 °C, with pH monitored every 3 h using a calibrated portable pH meter (HI8424, Hanna Instruments, Woonsocket, RI, USA). The time required to reach the target pH endpoint differed among substrates: pea flour acidified most rapidly (21 h), followed by lentil flour (22.5 h) and chickpea flour (23 h 30 min). Each substrate was immediately transferred to 4 °C upon reaching the target pH range, arresting further fermentation activity. This pH endpoint was considered indicative of complete lactic acid fermentation and a substantial reduction in antinutritional factors [14,15]. Following cooling, the fermented slurries were oven-dried at 50 °C to constant weight, ground, and sieved to recover a stabilised fermented legume flour—a form chosen to standardise moisture content, ensure precise control of ingredient proportions, and guarantee reproducibility during NutriCubes formulation [15,17]. The drying temperature of 50 °C was deliberately chosen to minimise thermal degradation of phenolic compounds and fermentation-derived volatile aroma constituents, which are susceptible to losses at temperatures above 60–70 °C [25].

### 2.3. NutriCubes Formulation and Processing

Preliminary qualitative observations during dough preparation indicated that legume flour proportions below 20% did not provide a meaningful nutritional contribution in terms of legume-derived protein, while proportions at or above 40% resulted in excessive dough stickiness that compromised processability. Based on these practical criteria, a ratio of 70:30 (oat:legume flour) was selected for all three NutriCubes formulations. Technologically, a 70% oat flour base provides the starch mass required for adequate gelatinisation during hot-water hydration and for maintaining dough cohesiveness during shaping [20,26].

A defining feature of the NutriCubes process was the use of hot water (85–90 °C) for dough preparation. Adding water at this temperature induces partial gelatinisation of oat starch granules in situ—a process that initiates around 53 °C and is substantially complete by 85 °C in oats [27]—without requiring a separate cooking step. The gelatinised starch matrix develops increased cohesiveness and plasticity, which facilitate clean cube formation by cutting, reduce crumbling during handling, and act as a structural binder during the subsequent drying step, contributing directly to the compact, dense texture of the finished product.

Three NutriCubes formulations were prepared (Table 1). Auxiliary ingredients were constant across all formulations.

The technological process was identical for all formulations: (i) dry ingredients were blended until homogeneous; (ii) oil was incorporated into the dry blend; (iii) hot water (85–90 °C) was added gradually at 32–36% of total dry flour blend weight (approximately 112 g per 200 g flour), with continuous stirring until a cohesive, non-sticky dough was obtained; (iv) the dough rested for 10–15 min to allow complete hydration and starch gelatinisation; (v) it was then portioned and cut manually into cubes of approximately 1 cm^3^; (vi) cubes were dried in a laboratory convection oven at 50 °C until constant weight, when a stable, crunchy texture was confirmed; and (vii) finished NutriCubes were cooled to ambient temperature and stored in sealed polyethylene bags pending analysis.

### 2.4. Methods

#### 2.4.1. Antinutritional Factors

Phytate content was determined spectrophotometrically by the Wade reagent method [28]: the ferric-sulfosalicylate complex (FeCl_3_ + sulfosalicylate) was displaced by phytate, and the decrease in absorbance at 500 nm was used to calculate phytate concentration, expressed as mg phytic acid/g DW. Phytate concentration was quantified against a phytic acid standard calibration curve constructed over a range of 0–50 mg phytic acid (y = −0.0106x + 0.6043; R^2^ = 0.9877).

Tannin content was determined by the Lowenthal-permanganate titrimetric method [29], in which an aqueous extract was titrated with standardised KMnO_4_ solution (0.117647 N) using indigo carmine as an indicator; results were expressed as mg tannic acid equivalent/g DW.

Both determinations were performed on unfermented and fermented legume flours to quantify the effect of the fermentation pre-treatment.

#### 2.4.2. In Vitro Protein Digestibility

In vitro protein digestibility (IVPD) was determined by sequential two-enzyme digestion [21]: 4 g of ground flour sample was suspended in 50 mL distilled water, pH adjusted to 2.0 with 6 N HCl, and 2 mL of pepsin solution (10% *w*/*v*; Sigma-Aldrich, St. Louis, MO, USA) added; the mixture was incubated at 37 °C for 2 h. The pH was then adjusted to 7.5 with 1 M NaOH, 5 mL of pancreatin solution (5% *w*/*v*; Sigma-Aldrich, USA) was added, and incubation continued at 37 °C for 2 h. Enzymes were inactivated by boiling for 10 min. After centrifugation (4500 rpm, 20 min, 15 °C), the residue was analysed for protein content and IVPD calculated as:

IVPD (%) = [(total protein − residue protein)/total protein] × 100(1)

#### 2.4.3. Proximate Composition

Moisture content was determined gravimetrically by drying at 105 ± 2 °C to constant weight (AOAC 930.15) [30]. Water activity (aw) of the finished NutriCubes was measured using a calibrated Rotronic hygrometer (INSTRUMART, Williston, VT, USA) based on dew point detection. The instrument was standardised prior to use with certified saline solutions to ensure measurement accuracy. Each sample was placed in a sealed chamber under controlled ambient conditions, and the aw reading was recorded after equilibrium was reached [31]. Protein content was measured by the Kjeldahl method (AOAC 920.87), using a nitrogen-to-protein conversion factor of 6.25. Total lipids were extracted by Soxhlet with petroleum ether (AOAC 920.39). Total ash was determined by incineration at 550 °C (AOAC 923.03). Total dietary fibre was quantified by the enzymatic-gravimetric method (AOAC 985.29) using the Megazyme Total Dietary Fibre Assay Kit (Megazyme International, Wicklow, Ireland) [30]. Available carbohydrates were calculated by difference. Energy was estimated using Atwater factors (protein × 4; carbohydrates × 4; lipids × 9 kcal/g) [32]. All proximate analyses were performed on both the raw flours (oat and fermented legume flours) and the finished NutriCubes formulations to evaluate compositional changes resulting from blending and thermal processing. All measurements were performed in triplicate.

#### 2.4.4. Amino Acid Profile and Protein Quality

The amino acid composition of the NutriCubes formulations was determined after protein hydrolysis with hydrochloric acid (6 N HCl, 105 °C, 24 h), followed by the identification and quantification of individual amino acids by HPLC-UV (AccQ·Tag method, Waters Corporation, Milford, MA, USA) [29]. Results were expressed as g amino acid per 100 g product and as g amino acid per 16 g nitrogen (g/16 g N).

The amino acid score (AAS) was calculated for each essential amino acid relative to the FAO/WHO/UNU (2007) reference pattern for adults [33]. An AAS value ≥ 100% indicates that the amino acid is not limiting relative to the reference pattern; the first limiting amino acid is the one with the lowest AAS.

The Protein Efficiency Ratio (PER) was estimated from the amino acid profile using the regression equations proposed by Kowalczewski et al. (2019) [34], which account for the specific amino acids that most influence protein quality for growth: leucine (Leu), proline (Pro), tyrosine (Tyr), methionine (Met), and histidine (His), expressed as g/16 g N:

(2) PER1 = −0.684 + 0.456 × Leu − 0.047 × Pro

(3) PER2 = −0.468 + 0.454 × Leu − 0.105 × Tyr

(4) PER3 = −1.816 + 0.435 × Met + 0.78 × Leu + 0.211 × His − 0.944 × Tyr

where Leu, Pro, Tyr, Met and His denote the amounts of the respective amino acids in g/16 g N. The three equations provide complementary estimates of PER—PER1 emphasises the leucine-proline balance, PER2 the leucine-tyrosine interaction, and PER3 integrates the contributions of methionine and histidine alongside leucine and tyrosine—and their mean is reported as the final PER value. Although these regression equations were originally calibrated on a pasta (wheat semolina) matrix [34], the amino acid input values (Leu, Pro, Tyr, Met, His) were those experimentally determined from the NutriCubes samples; the equations thus served as a validated predictive framework applied to product-specific compositional data. This approach represents a methodological limitation, as direct in vivo PER determination or PDCAAS calculation would provide greater matrix specificity. Equal weighting was applied as the most conservative approach, given that the three equations reflect complementary amino acid interactions and no matrix-specific validation data were available to justify differential weighting.

#### 2.4.5. Bioactive Compounds and Antioxidant Activity

Total polyphenol content (TPC) was determined by the Folin–Ciocalteu method [35]: methanolic extracts (80% *v*/*v*, 1:10 g/mL) were reacted with diluted Folin-Ciocalteu reagent and 7.5% Na_2_CO_3_, incubated at 40 °C for 30 min, and absorbance was read at 765 nm. Results were expressed as mg gallic acid equivalents per g dry weight (mg GAE/g DW). Antioxidant activity was assessed using the DPPH radical-scavenging method [33]: a 0.1 mM methanolic DPPH solution was mixed with the sample extract, and absorbance was measured at 517 nm after 30 min; results were expressed as the percentage inhibition relative to a blank. All measurements were performed in triplicate.

#### 2.4.6. Sensory Evaluation

Sensory evaluation was conducted with a panel of 107 assessors (students and academic staff of the Department of Food and Nutrition, Technical University of Moldova), enrolled in food science and nutrition programmes or employed in the field. Panellists ranged in age from 18 to 60 years (63% female, 37% male). Participation was voluntary; all participants provided written informed consent prior to evaluation. Individuals with known allergies to cereals or legumes, food intolerances, respiratory illness or cold at the time of evaluation, or smoking habits were excluded.

The study protocol was approved by the Departmental Ethics Committee. Sessions were conducted under controlled conditions (individual booths, natural daylight simulation, 20–22 °C). Samples were coded with three-digit random numbers and presented simultaneously in a randomised order. Each formulation was served as a 30 g portion, with water provided between samples to cleanse the palate. Each assessor evaluated all three formulations, presented sequentially in randomised order to minimise sensory fatigue.

Hedonic evaluation used a structured 9-point scale (1 = dislike extremely; 9 = like extremely) for eight attributes: appearance, colour, odour, taste, texture, aroma, aftertaste, and overall acceptability [36].

A Check-All-That-Apply (CATA) test was conducted in parallel, presenting panellists with a predefined list of 20 sensory descriptors (Table 2) from which they selected all terms applicable to each sample. CATA data were analysed by correspondence analysis and the Cochran’s Q-test for attribute significance [37].

### 2.5. Statistical Analysis

All measurements were performed in analytical triplicate on a single production batch per formulation; results are expressed as mean ± standard deviation. Differences between formulations for physicochemical and compositional parameters were assessed using one-way ANOVA, followed by Tukey’s HSD post hoc test (*p* < 0.05).

As the same assessors evaluated all three formulations, hedonic sensory data were analysed using one-way repeated-measures ANOVA, with formulation as the within-subject factor. Sphericity was assessed using Mauchly’s test; when the assumption of sphericity was violated, the Greenhouse–Geisser correction was applied. When a significant main effect of formulation was detected, Bonferroni-adjusted pairwise comparisons were performed, with statistical significance set at *p* < 0.05.

Pearson correlation analysis was used to explore relationships between compositional and sensory parameters. CATA data were analysed using correspondence analysis and Cochran’s Q test, with Marascuilo post hoc comparisons for pairwise attribute differences. All analyses were performed in SPSS Statistics v.26 (IBM, Armonk, NY, USA) and XLSTAT 2023 (Lumivero, Denver, CO, USA).

## 3. Results and Discussion

### 3.1. Proximate Composition of Raw Flours Before and After Lactic Fermentation

Before discussing the composition of the finished NutriCubes, it is worth examining what fermentation actually did to the legume flours themselves—because the ingredient that entered the formulation was not the commercially available flour but its fermented derivative. Table 3 reports the proximate composition of oat flour and the three legume flours (lentil, chickpea, pea) both before and after the lactic fermentation pre-treatment, expressed as a percentage of dry weight (g/100 g DM). The data reveal a consistent pattern of compositional change across all three legumes, though the magnitude varies considerably by species.

Lactic fermentation increased protein content in all three legume flours on a DM basis—most markedly in chickpea (+21.2%, from 23.92 to 29.00 g/100 g DM) and lentil (+13.2%, from 25.88 to 29.30 g/100 g DM), with a smaller gain in pea (+8.8%). Two mechanisms drive this pattern: LAB consume soluble carbohydrates as their primary carbon source, which reduces the total dry mass of the substrate and concentrates the protein fraction on a relative dry-weight basis, without necessarily implying an absolute increase in total nitrogen content; simultaneously, the proteolytic activity of *L. plantarum* and *L. casei* releases nitrogen-containing compounds previously bound in cell wall complexes, which are captured by the Kjeldahl method [39]. The more modest gain in pea protein likely reflects the predominantly globulin-structured nature of pea storage proteins and their lower susceptibility to LAB proteases [40]. Lipid content decreased slightly in all three legumes (−5.5% lentil, −10.8% chickpea, −21.1% pea), consistent with the lipolytic activity of LAB producing extracellular lipases that cleave triglycerides into free fatty acids subsequently used as microbial energy sources [16]. The disproportionately large percentage reduction in pea flour reflects its low absolute lipid content (1.91 g/100 g DM), where small absolute losses translate into large relative changes.

Total carbohydrate content decreased consistently (−5.4% lentil, −6.9% chickpea, −5.6% pea), driven by LAB consumption of soluble sugars and, potentially, by enzymatic hydrolysis of raffinose-family oligosaccharides (RFOs) via LAB-produced α-galactosidase—although RFOs were not directly quantified in the present study and this inference is based on the known α-galactosidase activity of the LAB strains used. If confirmed, the nutritional relevance of this would extend beyond the compositional change: RFOs—raffinose, stachyose, verbascose—are the principal cause of gastrointestinal discomfort that limits legume acceptance among many consumers, and their reduction would represent a genuine functional improvement in the fermented flour [41]. Dietary fibre decreased in all three legumes after fermentation (−17.3% lentil, −17.4% chickpea, −20.3% pea), reflecting partial microbial degradation of cell wall polysaccharides by LAB-produced glycosidases [16]. Despite this reduction, pea flour retained the highest fibre content after fermentation (17.52 g/100 g DM)—nearly double that of chickpea (10.62 g/100 g DM) and above oat flour (11.84 g/100 g DM). It is also worth noting that partial hydrolysis of insoluble fibre during fermentation may theoretically increase the soluble fibre fraction, which is not captured by the total dietary fibre assay; however, soluble and insoluble fibre fractions were not measured separately in the present study, and this remains an unverified inference that would contribute to prebiotic and glycaemic benefits [39].

### 3.2. Impact of Fermentation on Antinutritional Factors and In Vitro Protein Digestibility

**Figure 1:**
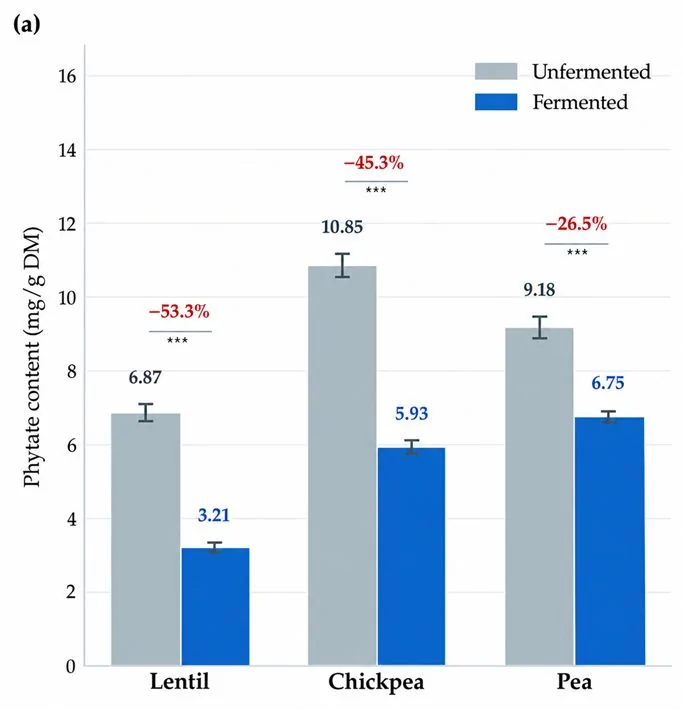

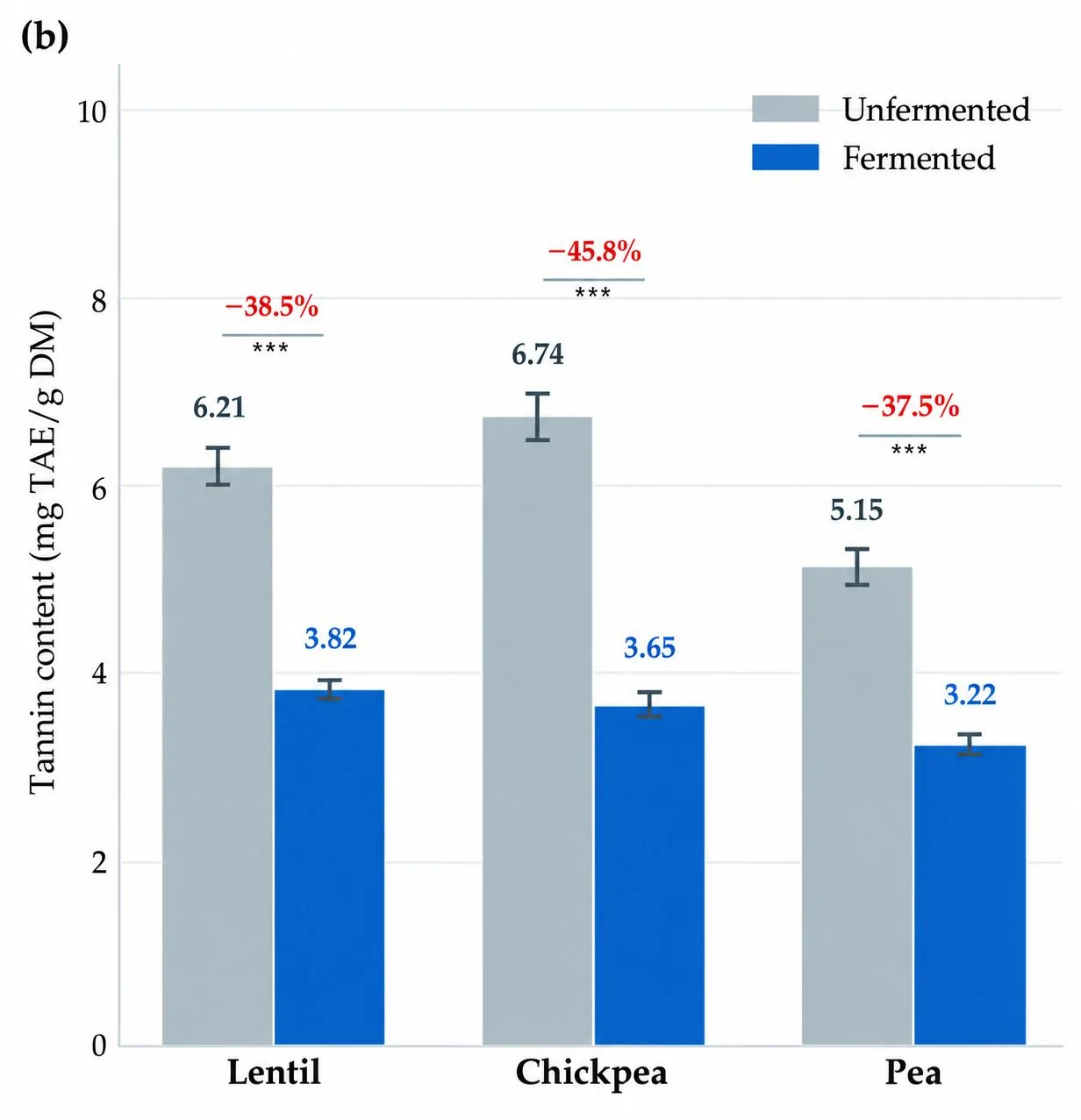

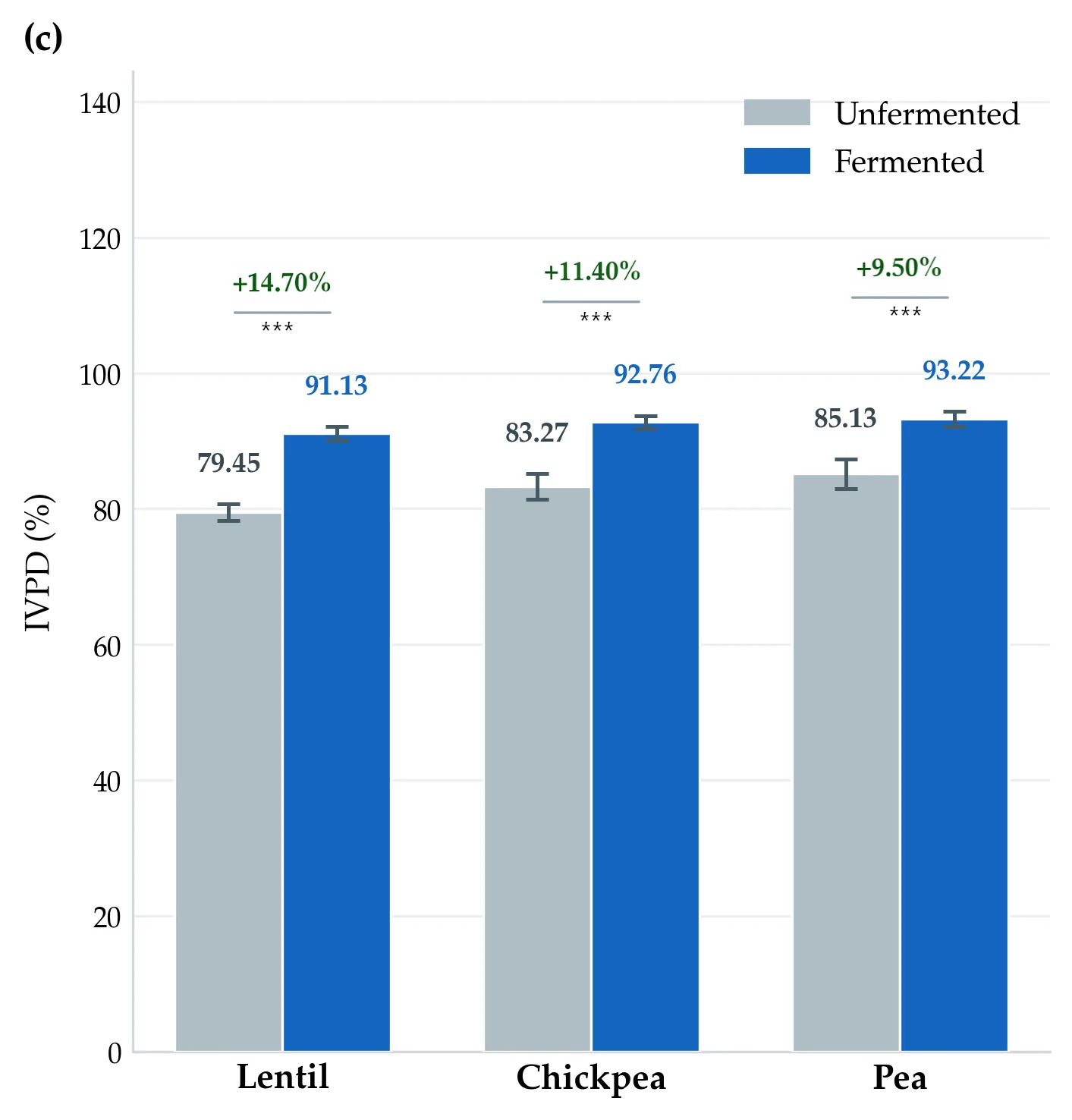
Effect of lactic acid fermentation on phytate content (**a**), tannin content (**b**), and in vitro protein digestibility—IVPD (**c**) of lentil, chickpea, and pea flours. *** *p* < 0.001 (paired *t*-test, fermented vs. unfermented within each legume).

The fermentation pre-treatment reduced both phytate and tannin content in all three legume flours and simultaneously improved in vitro protein digestibility—three outcomes that are connected rather than coincidental, since phytate and tannins each directly impair protein digestibility through different mechanisms: phytate forms stable complexes with digestive enzymes and minerals, while tannins crosslink proteins and reduce their susceptibility to proteolytic attack [15,16]. Kim et al. [42] recently reviewed the effect of legume fermentation on antinutritional factor reduction, highlighting that tannins, phytates, oligosaccharides, saponins and protease inhibitors are all responsive to LAB activity, with the magnitude of reduction depending on legume species, starter culture, and fermentation duration. The effect of the fermentation pre-treatment applied in the present study on phytate content, tannin content, and IVPD of the three legume flours is illustrated in Figure 1.

Phytate reductions were substantial: −53.3% in lentil (from 6.87 to 3.21 mg/g DM), −45.3% in chickpea, and −26.5% in pea. The phytase activity of the *L. plantarum*/*L. casei*/*L. delbrueckii* starter culture, amplified by the 35 °C tempering step applied before inoculation, which activates endogenous phytases in the legume matrix, is the primary driver of these reductions [15,40]. Byanju et al. reported phytate reductions of 7–42% in fermented lentil and green pea flours with *L. plantarum* and *P. acidilactici*, values somewhat lower than those observed here—a difference likely attributable to the three-strain mixed starter used in the present study. Senanayake et al. reviewed phytate reductions of 30–70% across LAB-fermented legume studies; the values obtained here fall within the lower-to-mid bound of this range, consistent with the short controlled fermentation applied (≤24 h to pH 4.5–4.6) [39]. Noori et al., working with solid-state fermentation of lentil and chickpea flours, similarly documented significant reductions in phytate alongside improved protein digestibility, confirming that these two effects are consistently coupled across different fermentation systems [43]. The more modest pea reduction (−26.5%) is consistent with observations that pea cell wall architecture limits the accessibility of substrates to microbial phytases [39,40].

Tannin content decreased by 38.5% in lentil (from 6.21 to 3.82 mg TAE/g DM), 45.8% in chickpea (from 6.74 to 3.65 mg TAE/g DM), and 37.5% in pea (from 5.15 to 3.22 mg TAE/g DM). Chickpea showed the largest absolute reduction, consistent with its higher initial tannin content. Tannin degradation during LAB fermentation is primarily mediated by tannase activity—an enzyme that hydrolyses ester and depside bonds in hydrolysable and condensed tannins—alongside the lower pH achieved during fermentation, which destabilises tannin-protein complexes and facilitates their degradation [16,44]. Álvarez et al. [44] documented tannin reductions of 15–80% in legume flours fermented with LAB under comparable conditions, a range that encompasses all three values obtained here. In a wider perspective, Arshad et al. [45] reported an 89% reduction in tannin content following LAB fermentation of grass pea flour—a comparatively extreme case, but illustrative of the upper boundary achievable by lactic fermentation when substrate and starter conditions are optimal. Sakandar et al. [14] reviewed the mechanistic basis of these reductions across multiple legume species, confirming that tannase-producing *L. plantarum* strains are among the most effective microorganisms for tannin degradation in legume matrices.

The improvement in in vitro protein digestibility was consistent across all three legumes: lentil +14.70%, pea + 9.50%, and chickpea + 11.40%. One result deserves a brief comment: the chickpea IVPD improvement (+9.49%) falls marginally below the literature range of 10–25% for LAB-fermented chickpea flours, though the absolute value reached (92.76%) is comparable to well-fermented chickpea matrices [39,46]. This slight underperformance relative to the lower bound is not altogether unexpected: chickpea storage proteins are dominated by 11S legumin-type globulins, which are more resistant to LAB proteases than the albumin-rich fractions prevalent in lentil, and its already-high unfermented IVPD (83.27%) leaves less room for improvement [14]. Senanayake et al. made the same observation, noting that chickpea tends to show lower relative IVPD gains per unit fermentation time compared to lentil or pea. The lentil result (+11.68%) aligns well with De Pasquale et al. [41], who documented gains of 8–15% in gelatinised lentil flours fermented with *L. plantarum*, while the pea improvement (+8.09%) is consistent with the 6–10% range reported by Skalickova et al. [47] for LAB-fermented pea flour. Noori et al. [43] confirmed that both phytate reduction and IVPD improvement occur simultaneously across lentil and chickpea matrices, supporting the view that these two effects are mechanistically linked: less phytate means fewer enzyme-phytate complexes and more active proteolysis during in vitro digestion.

A detail worth emphasising is that these outcomes were achieved simultaneously across three legume species in a single study—lentil, chickpea, and pea fermented under the same protocol and evaluated within the same product concept. Most published work on LAB fermentation of legumes focuses on one species at a time or varies fermentation conditions while keeping the legume fixed [39,43]. Here, the protocol was held constant and the legume varied, which means that the differences in phytate reduction, tannin degradation, and IVPD improvement observed across species can be attributed to the biological properties of each legume rather than to differences in processing—a distinction that adds interpretive value beyond what single-legume studies can offer.

### 3.3. Proximate Composition of NutriCubes Formulations

**Figure 2:**
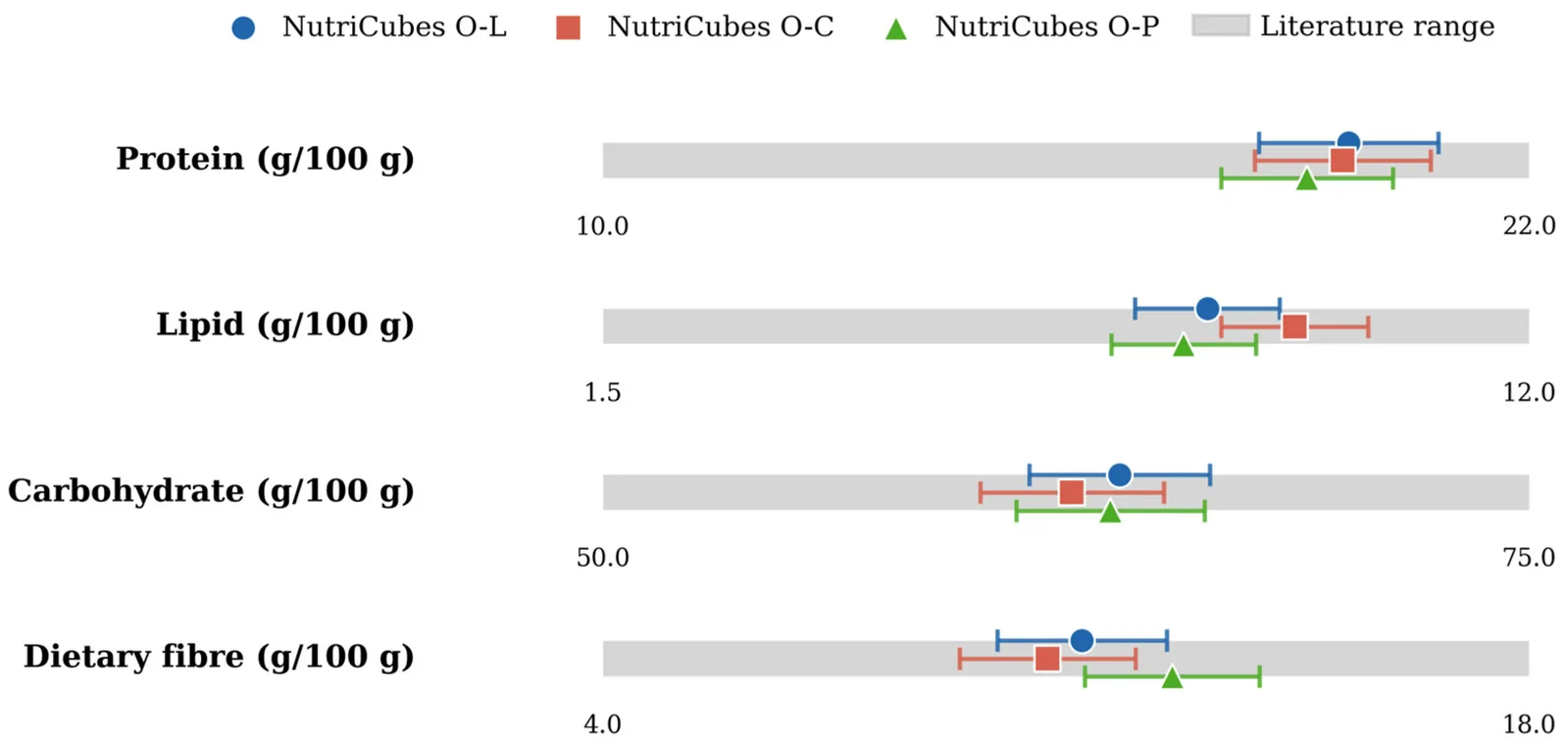
Proximate composition of NutriCubes formulations (O-L: Oat–Lentil; O-C: Oat–Chickpea; O-P: Oat–Pea) in relation to the range reported in the literature for cereal–legume baked and extruded snack products. Literature range compiled from [20,22,48,49,50,51].

The proximate composition of the three NutriCubes formulations, determined experimentally on the finished NutriCubes formulations using AOAC-validated methods, as described in Section 2.4.3, is illustrated in Figure 2. All formulations fall within the range reported in the literature for cereal–legume baked and extruded snack products across the major proximate parameters, with protein and dietary fibre contents positioned toward the upper end of that range. Moisture content was low across all samples (1.98–2.45 g/100 g FW), which is expected for thermally processed, shelf-stable snack products and measured water activity values ranging from 0.17 to 0.25 confirm microbiological stability, as both parameters fall well below the critical thresholds for microbial growth (aw < 0.60) and enzymatic activity (aw < 0.70), supporting the crunchy texture and shelf-life potential of the product.

Protein content was highest in O-L (19.66 g/100 g FW), followed closely by O-C (19.58 g/100 g FW) and O-P (19.12 g/100 g FW). Despite the narrow numerical differences, all three formulations fall in the upper portion of the 10–22 g/100 g range documented in the literature for cereal–legume snack products, which is a genuinely noteworthy result. For context, conventional corn-based extruded snacks typically contain around 6.9 g protein/100 g, and even fortified cereal–legume extrudates rarely exceed 15.5 g/100 g when legume substitution rates are kept below 50% [22,50]. The higher values recorded here reflect the relatively elevated legume-to-cereal ratio in the NutriCubes formulations. Among the legumes used, raw lentil flour has been reported to contain 25.33 g protein/100 g DM, while chickpea and pea flours provide 22.96 and 20.47–21.73 g/100 g DM, respectively [20], and comparable data are reported by Baik and Han (2012) for the same species [51]. The slightly lower protein content of O-P is consistent with this ranking, as pea generally shows the lowest protein among the three legumes when processed into flour.

Lipid content varied between 8.08 g/100 g FW (O-P) and 9.34 g/100 g FW (O-C), with O-L recording 8.35 g/100 g FW. The oat component is the primary contributor here, given that oats are considerably richer in fat than most other cereals [49]. The systematically higher lipid content of O-C is consistent with chickpea’s greater fat content relative to lentil and pea [22] and may also be influenced by the way fat redistributes during the non-extrusion thermal processing applied in NutriCubes production. These lipid levels, while not typical of low-fat extruded snacks, remain nutritionally reasonable and are largely attributable to unsaturated fatty acids from both oat and legume fractions [49].

Carbohydrate content was largely consistent across formulations, ranging from 62.66 g/100 g FW (O-C) to 63.94 g/100 g FW (O-L), with O-P at 63.69 g/100 g. The slightly lower value in O-C mirrors the known carbohydrate profile of chickpea, which provides a lower fraction of available carbohydrates (~50 g/100 g) compared to lentil and pea [22]. All three values fall in the mid-to-upper portion of the 50–75 g/100 g literature range and are comparable to those reported for similar cereal–legume dried or baked snack products (67–70.5 g/100 g) [50]. It is also worth noting that legume-derived carbohydrates include a significant resistant starch and oligosaccharide fraction that is less rapidly digested than cereal starch—a characteristic that may confer beneficial glycaemic properties to the final product [51].

Dietary fibre content followed a clear ranking: O-P > O-L > O-C, with values of 12.60, 11.24, and 10.72 g/100 g FW, respectively. These results place all three formulations comfortably within the middle of the 4–18 g/100 g range reported in the literature [48], and are noteworthy from a functional food perspective—a 100 g serving of any NutriCubes variant would cover approximately 36–43% of the daily fibre intake recommended by FAO/WHO (25–30 g/day). The higher fibre content of O-P is in line with peas’ recognised richness in both soluble and insoluble dietary fibres, while the oat fraction contributes β-glucan, a soluble fibre with well-documented cholesterol-lowering and prebiotic effects. The lower fibre content of O-C, though still nutritionally significant, reflects the fact that chickpea tends to yield less total dietary fibre in processed products compared to lentil- or pea-enriched matrices [48].

### 3.4. Amino Acid Profile of NutriCubes Formulations

The decision to combine oat flour with fermented legume flours was not purely technological—it was nutritionally motivated. Cereal proteins are well known to fall short in lysine while covering sulphur amino acid requirements adequately; legume proteins do the opposite, supplying abundant lysine but limited methionine and cysteine [8,33]. At a 70:30 blend ratio, the two ingredient groups correct each other’s deficiencies, and the amino acid profile of the finished NutriCubes reflects this mutual compensation. The amino acid composition is presented in Table 4.

Total amino acid content ranged from 14.76 g/100 g FW (O-P) to 15.79 g/100 g FW (O-L), with essential amino acids accounting for 35.4–36.5% of total amino acids across formulations, approaching the 40% threshold historically considered indicative of good protein quality [52]. A more rigorous evaluation against the FAO/WHO/UNU (2013) reference pattern is provided through the AAS analysis reported in Table 4. Although numerical variations in individual amino acid concentrations were observed among O-L, O-C, and O-P, none of these differences reached statistical significance (*p* > 0.05).

Lysine was particularly relevant in the context of cereal–legume protein complementation. Its concentration ranged from 0.799 to 0.856 g/100 g FW across the three formulations, exceeding the value reported for oat flour alone (0.63 g/100 g) and suggesting a meaningful contribution of the legume fraction to the lysine content. Wani et al. [26] reported a similar pattern in oat–pea extruded snacks, where pea flour raised lysine content well above cereal-only references; Ziena and Ziena [22] found lysine to be the first limiting amino acid in corn-based control snacks, with values nearly doubling after legume incorporation. Nevertheless, when evaluated against the FAO/WHO/UNU (2013) reference pattern, lysine remained the first limiting amino acid in O-L (AAS = 96.8%) and O-C (AAS = 90.7%). In O-P, lysine also remained below the reference pattern (AAS = 92.9%), although valine and isoleucine showed even lower and nearly identical AAS values (82.5 and 82.6%, respectively).

Although methionine had the lowest absolute concentration among the individually quantified essential amino acids (0.212–0.224 g/100 g FW), protein quality assessment against the FAO/WHO/UNU reference pattern considers methionine together with cysteine. The combined Met+Cys AAS ranged from 129.1 to 139.5%, indicating that sulphur-containing amino acids were not limiting in any of the formulations. Overall, these findings illustrate the complementary nature of cereal and legume proteins: the legume fraction contributed to improving the lysine content of the oat-based matrix, whereas the oat fraction contributed to an adequate supply of sulphur-containing amino acids, consistent with observations by López-Martínez et al. [21]. In terms of absolute concentration, leucine was the most abundant essential amino acid across all formulations (1.083–1.257 g/100 g FW), followed by lysine and valine - a profile characteristic of cereal–legume blends and not without metabolic relevance, given leucine’s documented role in stimulating muscle protein synthesis [53]. Among the non-essential amino acids, glutamic acid dominated (2.900–3.238 g/100 g FW), consistent with its abundance in the constituent cereal and legume proteins.

Finally, fermentation may increase the free amino acid pool through the proteolytic activity of lactic acid bacteria [54]. Such changes could potentially enhance amino acid accessibility and protein digestibility; however, these effects cannot be inferred directly from total amino acid analysis and would require specific assessment of free amino acids and protein digestibility.

### 3.5. Protein Efficiency Ratio (PER) of NutriCubes Formulations

The amino acid composition data discussed above provide a compositional overview, but protein quality is ultimately about the ability of a protein to support growth and maintenance—which depends not only on which amino acids are present but also on their relative proportions. The Protein Efficiency Ratio (PER) was estimated using the three complementary regression equations of Kowalczewski et al. [55]—PER1 (leucine-proline balance), PER2 (leucine-tyrosine interaction), and PER3 (methionine, leucine, histidine and tyrosine combined)—whose arithmetic mean is reported as the final PER value, providing a more robust estimate than any single equation alone. It should be noted that these are predicted PER values derived from amino acid composition and have not been validated through experimental animal feeding trials; predicted and experimentally determined PER values may not be directly comparable. The resulting PER values are presented in Table 5.

The ranking—O-L (1.989 ± 0.293) > O-P (1.754 ± 0.298) > O-C (1.510 ± 0.276)—indicates a numerical trend that reflects differences in leucine content rather than any intrinsic superiority of one legume over another. For each individual PER equation (PER1–PER3), no significant differences were observed among the three formulations (*p* > 0.05). Leucine drives two of the three equations, and O-L simply delivers more of it relative to its protein fraction than O-C. PER3, which brings methionine into the picture, was consistently the lowest of the three estimates across all formulations—an expected outcome given that methionine is the classic limiting amino acid of legume proteins, and the values obtained here are typical for cereal–legume blends at this substitution level [8].

### 3.6. Total Polyphenol Content and Antioxidant Activity of NutriCubes

Beyond protein and fibre, polyphenols represent a third nutritional dimension of the NutriCubes formulations—one shaped not only by the legume flour used but also by the fermentation pre-treatment, which releases bound phenolics from cell-wall matrices and increases their extractability [56]. The total polyphenol content (TPC) and DPPH radical scavenging activity, both determined by methanol extraction, are presented in Table 6.

The results placed O-L clearly above the other two formulations, with 6.28 ± 0.13 mg GAE/g DM and 72.9 ± 1.22% DPPH inhibition, consistent with lentil being documented as the most polyphenol-rich of the three legumes used here [56,57]. O-P and O-C followed at 5.16 ± 0.09 mg GAE/g DM (51.9 ± 2.43% DPPH) and 4.32 ± 0.16 mg GAE/g DM (44.6 ± 1.31% DPPH), respectively. All three values fall within the 1.16–7.45 mg GAE/g DW range reported for cooked Canadian pulses [58], and the strong agreement between TPC ranking and DPPH ranking reflects the well-established correlation between these two parameters in legume matrices—a relationship confirmed here by Pearson correlation analysis across all formulations and replicates (r = 0.976, *p* < 0.0001, *n* = 9), indicating that phenolic compounds are the primary drivers of antioxidant capacity in NutriCubes. The lower antioxidant activity of O-C is not surprising: kabuli-type chickpea varieties, which have a thin, pale seed coat, are consistently among the lower-polyphenol legumes compared to darker-seeded lentil varieties [56,57,59].

One aspect of the NutriCubes polyphenol profile that total TPC values do not capture is the contribution of oat avenanthramides—phenolic alkaloids unique to oats, not present in any of the three legume flours—which have documented anti-inflammatory and antiatherogenic properties independent of their DPPH scavenging capacity [60]. This means that the antioxidant functionality of NutriCubes, particularly O-L, is likely broader than what the DPPH assay alone conveys, and that complementary assays such as ABTS or ORAC would provide a more complete picture of the antioxidant profile—something worth considering for future work.

### 3.7. Sensory Evaluation

#### 3.7.1. Hedonic Evaluation of NutriCubes Formulations

**Figure 3:**
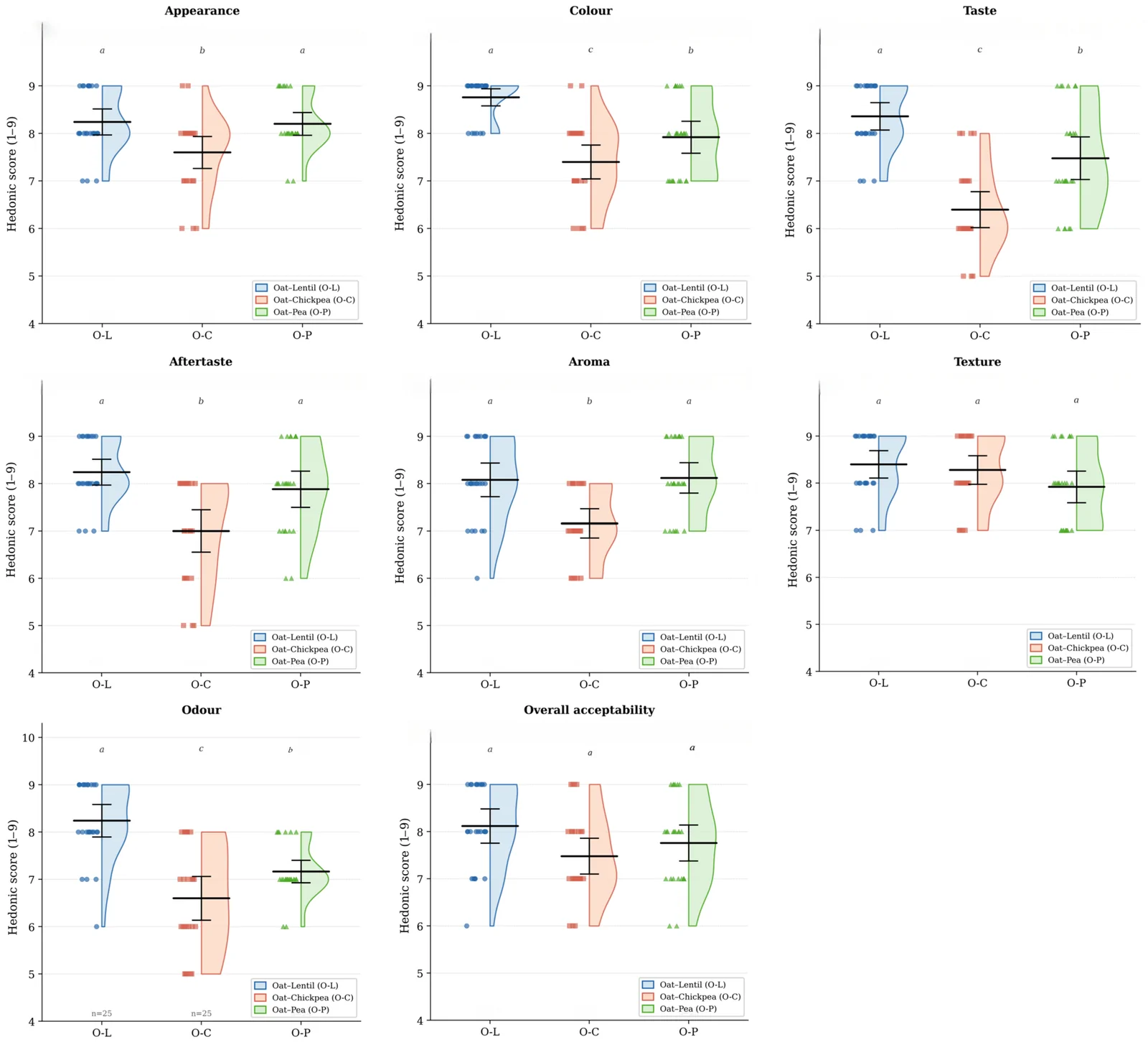
Violin plots showing the distribution of hedonic scores for eight sensory attributes of NutriCubes formulations (O-L: Oat–Lentil; O-C: Oat–Chickpea; O-P: Oat–Pea) assessed by a hedonic panel (*n* = 107). The horizontal line indicates the mean; vertical bars represent the 95% confidence interval. Individual data points correspond to scores assigned by each panellist. Different lowercase letters indicate significant differences between formulations based on Bonferroni-adjusted pairwise comparisons following repeated-measures ANOVA (*p* < 0.05).

The sensory acceptability of the three NutriCubes formulations was assessed by a hedonic panel of 107 evaluators across seven attributes. The distribution of individual scores, visualised as violin plots overlaid with individual data points and the mean ± 95% CI, is shown in Figure 3.

All three NutriCubes formulations were well accepted, with overall acceptability scores ranging from 7.48 (O-C) to 8.12 (O-L) on the 9-point hedonic scale—all above the ‘like slightly’ threshold. O-L and O-P were not significantly different, while O-C scored lower, with O-P intermediate. These results compare favourably with scores reported for legume-based snacks by López-Martínez et al. [21] (7.8/9) and Sattar et al. [61] (7.1–7.3/9).

Taste showed the greatest differentiation: O-L scored highest (8.36 ± 0.70), O-P was intermediate (7.52 ± 0.92), and O-C was lowest (6.40 ± 0.87). The lower score for O-C reflects the characteristic beany off-notes of chickpea flour, which may be driven by lipoxygenase-derived volatile compounds, documented as a primary barrier to chickpea snack acceptance [6,62], which persist even after lactic fermentation [63].

Texture showed no significant differences between formulations (all 7.96–8.28), confirming that the processing protocol produced a uniformly crunchy matrix regardless of legume type [63]. This contrasts with extrusion-based studies, in which lentil flour produced harder products than pea-based equivalents [64].

Colour showed the greatest differentiation among all attributes, with all three groups differing significantly (O-L: 8.76 ± 0.44, O-P: 7.92 ± 0.64; O-C: 7.40 ± 0.87). The superior score of O-L reflects pronounced Maillard browning of lentil flour, which may be driven by its high lysine content [65,66], while the paler surface of O-C is consistent with the lower reducing sugar content of chickpea flour [6].

Aroma, appearance, and aftertaste followed analogous patterns, with O-C consistently scoring lower than O-L and O-P, likely due to residual lipoxygenase-derived aldehydes that were only partially attenuated by fermentation [15]. Odour scores showed a similar pattern and differed significantly among formulations (O-L: 8.24 ± 0.83; O-P: 7.17 ± 0.56; O-C: 6.60 ± 1.12), with O-L receiving the highest ratings, consistent with the more pleasant volatile profile associated with lactic fermentation of lentil flour. O-P occupied an intermediate sensory position across all attributes and represents a nutritionally and sensorially viable alternative to O-L.

#### 3.7.2. CATA Sensory Characterisation

**Figure 4:**
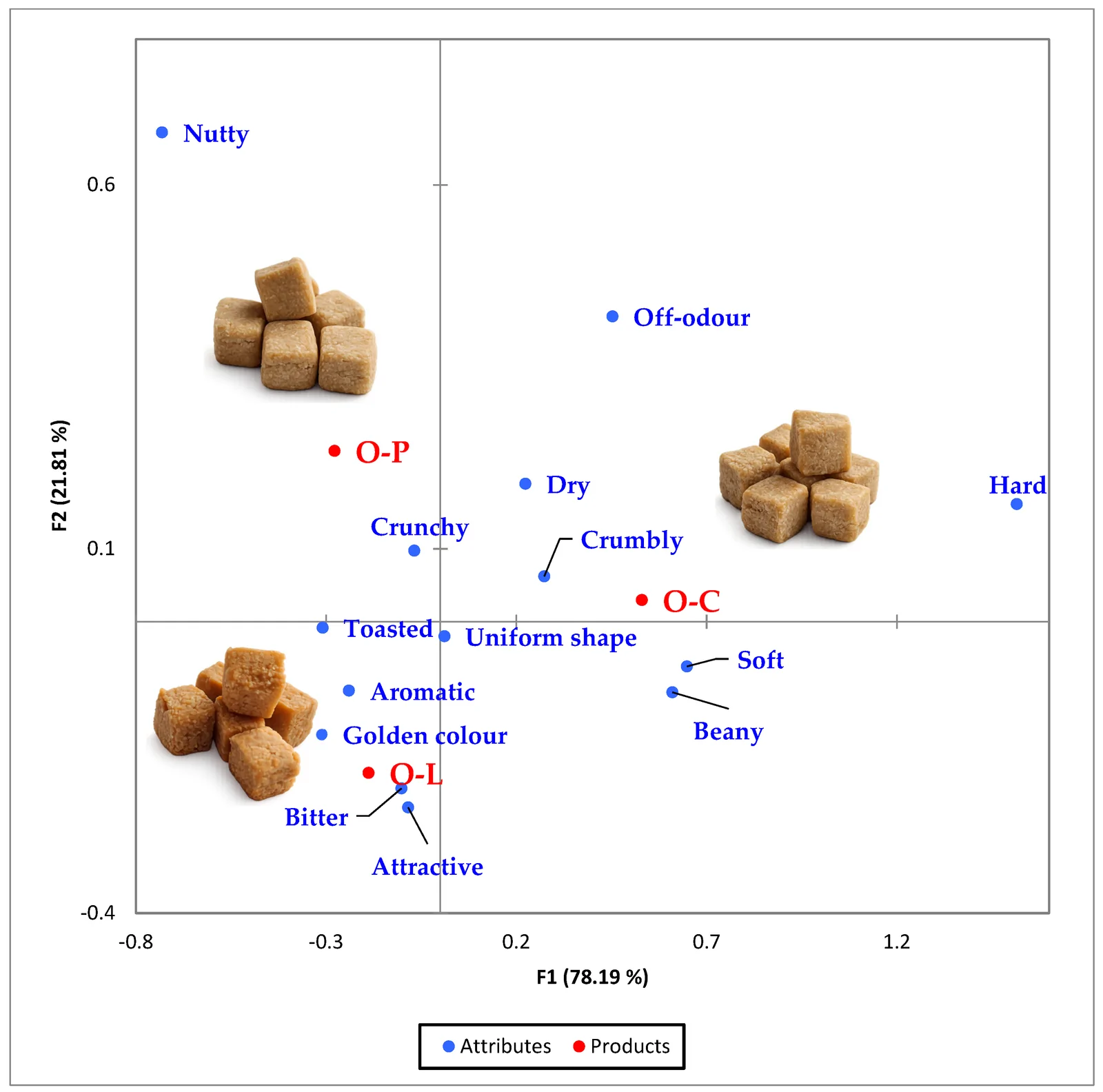
Correspondence Analysis (CA) symmetric biplot of CATA attribute frequencies for NutriCubes formulations (*n* = 107).

While the hedonic scores quantified how much panellists liked each formulation, the CATA task asked them to describe what they actually perceived—translating individual sensory impressions into a collective fingerprint for each product. Cochran’s Q test identified significant differences among the three NutriCubes formulations for 10 of the 20 attributes evaluated (*p* < 0.05), with Marascuilo post hoc comparisons used to identify pairwise differences [67,68]. All assessors evaluated all three formulations, satisfying the complete-block assumption of the test. Attributes with proportions below 0.20 were interpreted with caution given their sensitivity to sampling variability. Attributes with zero citation frequency across all three formulations were excluded from CA because they provided no discriminatory information. The Correspondence Analysis (CA) perceptual map is shown in Figure 4.

The correspondence analysis (CA) biplot (Figure 4) accounts for 100% of the total inertia in two dimensions (χ^2^ = 275.598, df = 26, *p* < 0.0001), with F1 (78.19%) representing the primary axis of product differentiation and F2 (21.81%) primarily discriminating between O-P and O-L.

F1 clearly separates O-C (positive side) from O-L and O-P (negative side). O-C is characterised by Beany (proportion = 0.757, the highest among products, *p* < 0.001) and Hard (0.243 vs. 0 for both O-L and O-P, *p* < 0.001), which were the two largest contributors to F1 (24.3% and 27.7%, respectively). Dry was also more frequently cited for O-C (0.636) than for O-L (0.402) and O-P (0.505), although this difference did not reach statistical significance (*p* = 0.053). The pronounced beany character of O-C is consistent with the well-documented persistence of chickpea-derived volatile compounds—in particular, hexanal and other lipid oxidation products—that survive thermal processing [69]. The Hard texture, unique to O-C, contrasts with the universally Crunchy profile shared by O-L and O-P (both 1.000, *p* < 0.001), and may reflect the higher protein content of chickpea flour, which restricts starch gelatinisation and structural expansion during thermal processing, resulting in a denser, less friable matrix [3].

O-L and O-P are positioned together on the negative side of F1, sharing higher citation frequencies for Crunchy (both 1.000), Aromatic, and Toasted than O-C. The Aromatic (O-L: 0.822; O-P: 0.561) and Toasted (O-L: 0.701; O-P: 0.579) attributes are consistent with fermentation-derived volatile compounds—including aldehydes, esters, and organic acids—produced during lactic acid fermentation of the plant matrix [46,70].

F2 discriminates between O-P (positive F2) and O-L (negative F2), with the two formulations contributing almost equally to this dimension (49.7% and 49.5%, respectively). O-P is distinctly associated with Nutty (0.360 vs. 0.120 for O-L, and 0 for O-C; the highest value and significantly different from both other products, *p* < 0.001), which was the dominant contributor to F2 (39.1%) and may reflect pyrazines and aldehyde-type compounds characteristic of pea-derived ingredients naturally present in fermented pea flour [69,71,72]. O-L achieved the highest score for Golden colour (0.680 vs. 0.200 for O-C and 0.420 for O-P), and was also positioned in the direction of Attractive (0.920), Aromatic (0.820), and Golden colour on the CA map. Attractive was the second-largest contributor to F2 (20.4%), supporting the sensory differentiation of O-L from O-P along this dimension. The characteristic amber-golden hue of the lentil-oat dried matrix constitutes a strong visual quality cue associated with perceived quality in cereal-based snack products [73].

It is also worth noting that the sensory approach used here goes somewhat further than what is typical in this product category, where hedonic scores from panels of 20–50 evaluators remain the norm [21,61]. The addition of a 20-descriptor CATA test across 107 panellists moves beyond aggregate liking to reveal which specific attributes drive acceptance or rejection for each formulation—a distinction that the perceptual map makes immediately actionable: *Beany* and *Hard* emerge as the primary barriers to O-C adoption, a finding that no hedonic score alone would have surfaced.

## 4. Conclusions

This study demonstrates that lactic acid fermentation is an effective pre-treatment for legume flours used in snack production. Fermentation with a mixed LAB starter at 30 °C reduced phytate by 26.5–53.3% and tannins by 37.5–45.8% across lentil, chickpea, and pea flours. These reductions fall within, and in some cases at the upper end of, the ranges reported for LAB fermentation and are associated with enhanced protein digestibility and a likely improvement in mineral bioaccessibility. These outcomes translated directly into the nutritional profile of the finished NutriCubes: protein contents of 19.12–19.66 g/100 g FW and dietary fibre of 10.72–12.60 g/100 g FW positioned all three formulations above the range typical of conventional cereal-based snacks, alongside a complementary amino acid balance and Protein Efficiency Ratios consistent with nutritionally acceptable plant-based products.

From a bioactive standpoint, O-L led across all parameters (6.28 mg GAE/g DM; 72.9% DPPH inhibition), consistent with the known phenolic profiles of oat and lentil, including avenanthramides and flavonoids, whose extractability is enhanced by fermentation. Consumer evaluation confirmed that this nutritional advantage extended to sensory performance: O-L scored highest for overall acceptability (8.12/9), while the oat–pea variant (O-P) represented a consistently well-accepted alternative. The oat–chickpea formulation (O-C), despite its nutritional merit, was limited by persistent beany off-notes—a constraint that would require targeted mitigation before commercial deployment.

These findings establish NutriCubes as a nutritionally sound and technically feasible low-temperature dried snack format, with the oat–lentil variant representing the most promising formulation across nutritional, bioactive, and sensory dimensions. Future work should address scale-up feasibility, shelf-life evaluation under different packaging conditions, the glycaemic index, DIAAS determination, mineral bioavailability, and long-term safety assessment, as well as the regulatory positioning of NutriCubes relative to existing protein and fibre labelling thresholds. A key methodological limitation of the present study is that compositional, antinutrient, amino acid, PER, and bioactive analyses were performed in analytical triplicate on a single production batch per formulation (biological *n* = 1). Consequently, the ANOVA/Tukey HSD comparisons reflect analytical rather than biological replication, and the statistical differences reported should be interpreted with caution. Future studies should employ independent biological replicates (separate fermentation and production batches) to confirm the reproducibility of the observed differences.

## Figures and Tables

**Table 1:** NutriCubes formulations (per 200 g total flour blend).

Ingredient	O-L	O-C	O-P
Oat flour (g)	140	140	140
Fermented legume flour (g)	60 (lentil)	60 (chickpea)	60 (pea)
Hot water * (g)	112	112	116
Sunflower oil (g)	5	5	5
Salt (g)	3	3	3
Dried onion (g)	2	2	2
Dried garlic (g)	0.6	0.6	0.6
Mustard powder (g)	1	1	1

O-L (Oat:Lentil 70:30), O-C (Oat:Chickpea 70:30), O-P (Oat:Pea 70:30). * Water was added at 85–90 °C (32–36% of total dough mass, adjusted individually per formulation based on dough consistency) to induce partial in situ gelatinisation of oat starch.

**Table 2:** Sensory descriptors used in the Check-All-That-Apply (CATA) test for NutriCubes evaluation (*n* = 20 descriptors).

Category	Descriptor	Definition/Sensory Anchor
Texture	Crunchy	Hard, brittle resistance when bitten
	Hard	Requires considerable force to bite through
	Soft	Little resistance when bitten
	Crumbly	Breaks into small pieces easily
	Dry	Low moisture perception in mouth
Flavour/Taste	Salty	Perceivable salt sensation
	Nutty	Reminiscent of nuts or roasted seeds
	Beany	Characteristic raw legume flavour
	Bitter	Bitter sensation on palate
	Bland	Absence of pronounced flavour
	Spicy	Perceivable spice note (mustard/garlic)
	Sour	Acid/sour sensation—fermentation note
Aroma	Aromatic	Pleasant, complex aroma
	Grainy	Cereal/oat characteristic aroma
	Toasted	Baked, roasted aroma note
	Off-odour	Unpleasant or atypical odour
Appearance	Golden colour	Uniform golden-yellow surface colour
	Dark colour	Brown to dark brown surface colour
	Uniform shape	Regular cube shape, consistent dimensions
	Attractive	Overall visually appealing

Descriptors were selected based on a preliminary focus group (*n* = 8) and adapted and refined based on published CATA lists for cereal–legume snack products [37,38].

**Table 3:** Proximate composition of oat flour and lentil, chickpea, and pea flours before (unfermented) and after lactic fermentation (g/100 g DM, mean ± SD, *n* = 3).

Parameter	Oat Flour	Lentil Flour		Chickpea Flour		Pea Flour	
		Unfermented	Fermented	Unfermented	Fermented	Unfermented	Fermented
Moisture (g/100 g FM)	11.96 ± 0.23 ^bc^	10.31 ± 0.46 ^a^	11.12 ± 0.24 ^ab^	13.34 ± 0.17 ^d^	13.45 ± 0.32 ^d^	10.44 ± 0.26 ^a^	11.68 ± 0.28 ^c^
Protein	18.11 ± 1.70 ^a^	25.88 ± 1.24 ^bc^	29.30 ± 1.49 ^c^	23.92 ± 2.03 ^b^	29.00 ± 1.12 ^c^	25.26 ± 1.74 ^bc^	27.49 ± 0.84 ^bc^
Lipids	7.84 ± 1.27 ^c^	2.63 ± 0.23 ^ab^	2.49 ± 0.10 ^ab^	6.54 ± 1.19 ^c^	5.83 ± 0.36 ^c^	1.91 ± 0.64 ^ab^	1.51 ± 0.12 ^a^
Carbohydrates	71.90 ± 3.69 ^b^	69.70 ± 1.72 ^b^	65.97 ± 2.39 ^ab^	64.89 ± 4.47 ^ab^	60.43 ± 2.59 ^a^	69.26 ± 3.49 ^b^	65.40 ± 3.44 ^ab^
Dietary fibre	11.84 ± 1.93 ^a^	15.22 ± 0.81 ^ab^	12.58 ± 0.95 ^a^	12.85 ± 2.77 ^ab^	10.62 ± 1.46 ^a^	21.97 ± 2.01 ^c^	17.52 ± 1.48 ^bc^

Values are presented as mean ± SD (*n* = 3). Different superscript letters within the same row indicate significant differences according to Tukey’s HSD test (*p* < 0.05). Carbohydrates include dietary fibre. Moisture is reported on a fresh weight basis (g/100 g FW); all other components are expressed on a dry matter basis (g/100 g DM).

**Table 4:** Amino acid composition of NutriCubes formulations (g/100 g FW, mean ± SD; *n* = 3).

Amino Acid	FAO/WHO Standard (2013), mg/g [52]	O-L, g/100 g FW	g Amino Acid/100 g Protein	AAS, %	O-C, g/100 g FW	g Amino Acid/100 g Protein	AAS, %	O-P, g/100 g FW	g Amino Acid/100 g Protein	AAS, %
Aspartic acid		1.586 ± 0.163			1.492 ± 0.174			1.523 ± 0.177		
Glutamic acid		3.192 ± 0.261			3.238 ± 0.296			2.900 ± 0.266		
Alanine		0.796 ± 0.096			0.772 ± 0.095			0.723 ± 0.091		
Arginine		1.170 ± 0.129			1.313 ± 0.151			1.120 ± 0.132		
Cysteine		0.361 ± 0.050			0.344 ± 0.050			0.371 ± 0.051		
Phenylalanine *	-	0.863 ± 0.100			0.897 ± 0.102			0.804 ± 0.094		
Glycine		0.801 ± 0.096			0.767 ± 0.092			0.767 ± 0.092		
Histidine *	15	0.393 ± 0.045	2.00	133.3	0.391 ± 0.045	2.00	133.1	0.451 ± 0.051	2.36	157.3
Isoleucine *	30	0.633 ± 0.073	3.22	107.3	0.605 ± 0.070	3.09	103.0	0.474 ± 0.060	2.48	82.6
Leucine *	59	1.257 ± 0.140	6.39	108.4	1.083 ± 0.134	5.53	93.7	1.177 ± 0.139	6.16	104.3
Lysine *	45	0.856 ± 0.091	4.35	96.8	0.799 ± 0.091	4.08	90.7	0.799 ± 0.091	4.18	92.9
Methionine *	-	0.224 ± 0.029	-	-	0.212 ± 0.028	-	-	0.216 ± 0.028	-	-
Methionine * + Cysteine	22		2.98	135.3		2.84	129.1		3.07	139.5
Proline		0.746 ± 0.089	-	-	0.778 ± 0.090	-	-	0.800 ± 0.092	-	-
Serine		0.882 ± 0.102	-	-	0.833 ± 0.097	-	-	0.792 ± 0.094	-	-
Tyrosine		0.489 ± 0.058	-	-	0.477 ± 0.057	-	-	0.541 ± 0.062	-	-
Phenylalanine * + Tyrosine	38		6.88	181.0		7.02	184.7		7.03	185.1
Threonine *	23	0.598 ± 0.070	3.04	132.2	0.519 ± 0.062	2.65	115.2	0.547 ± 0.065	2.86	124.4
Tryptophan *	6	0.124 ± 0.016	0.63	105.1	0.127 ± 0.016	0.65	108.1	0.140 ± 0.017	0.73	122.0
Valine *	39	0.815 ± 0.099	4.15	106.3	0.767 ± 0.094	3.92	100.4	0.615 ± 0.085	3.22	82.5
Total EAA *		5.763			5.400			5.223		
Total amino acids		15.786			15.414			14.760		

O-L (Oat:Lentil 70:30), O-C (Oat:Chickpea 70:30), O-P (Oat:Pea 70:30). No significant differences were observed among formulations for individual amino acids (Tukey’s HSD test, *p* > 0.05). * Essential amino acids.

**Table 5:** Predicted PER values for NutriCubes formulations based on amino acid composition

Parameter	O-L	O-C	O-P
PER1	2.053 ± 0.168 ^b^	1.651 ± 0.046 ^a^	1.926 ± 0.070 ^b^
PER2	2.174 ± 0.068 ^b^	1.787 ± 0.145 ^a^	2.030 ± 0.070 ^b^
PER3	1.741 ± 0.107 ^c^	1.091 ± 0.270 ^a^	1.304 ± 0.025 ^b^
PER mean	1.989 ± 0.093 ^c^	1.510 ± 0.076 ^a^	1.754 ± 0.198 ^b^

O-L (Oat:Lentil 70:30), O-C (Oat:Chickpea 70:30), O-P (Oat:Pea 70:30). a, b, c Different superscript letters within a row indicate statistically significant differences among formulations (one-way ANOVA, Tukey’s HSD post hoc test, *p* < 0.05). O-C exhibited the lowest predicted PER values across all three equations, differing significantly from both O-L and O-P.

**Table 6:** Total polyphenol content (TPC) and DPPH radical scavenging activity of NutriCubes formulations (mean ± SD; *n* = 3).

Formulation	TPC (mg GAE/g DM)	DPPH Inhibition (%)
O-L	6.28 ± 0.13 ^c^	72.9 ± 1.22 ^c^
O-P	5.16 ± 0.09 ^b^	51.9 ± 2.43 ^b^
O-C	4.32 ± 0.16 ^a^	44.6 ± 1.31 ^a^

O-L (Oat:Lentil 70:30), O-C (Oat:Chickpea 70:30), O-P (Oat:Pea 70:30). Values are expressed as mean ± SD (*n* = 3). Different superscript letters within the same column indicate significant differences among formulations according to Tukey’s HSD test (*p* < 0.05).

## Data Availability

The original contributions presented in the study are included in the article, further inquiries can be directed to the corresponding author.
